# Emergence of Multidrug-Resistant Pathogens in a Tertiary Care Hospital in Northern India: A Comparative Study Between Critical Care Units and Wards

**DOI:** 10.7759/cureus.107448

**Published:** 2026-04-21

**Authors:** Manish Kumar, Ranga Reddy Burri, Shalabh Jauhari

**Affiliations:** 1 Microbiology, Government Doon Medical College, Dehradun, IND; 2 Medicine and Public Health, University of Hyderabad, Hyderabad, IND

**Keywords:** antimicrobial sensitivity, device-related infection, immunocompromised patient, multidrug-resistant organisms (mdros), prior antibiotic exposure, universal precautions

## Abstract

Background and aim

Multidrug-resistant organisms (MDROs) represent a major global public health concern, largely driven by inappropriate antibiotic use, over-the-counter availability of antimicrobials, and the absence of effective prescription audit systems. MDROs are microorganisms, most commonly bacteria, that exhibit resistance to one or more classes of antimicrobial agents. The burden of multidrug-resistant infections has increased worldwide, with marked rises in both healthcare-associated and community-acquired infections, particularly in regions with high levels of antibiotic misuse. This study aimed to characterize the spectrum of MDROs circulating in a tertiary care center, identify associated risk factors, and describe their antibiotic susceptibility patterns.

Methods

A prospective observational study was conducted over a period of one month, involving 1,223 clinical specimens from predefined areas. Specimens were processed for culture, identification, and antibiotic susceptibility testing. For all samples, patient demographic details, clinical information, and risk factors were documented and assessed. Infection prevention and control-related information was also documented. Data were analyzed using IBM SPSS Statistics for Windows, version 22.0 (released 2013; IBM Corp., Armonk, NY, USA), with the incorporation of the chi-square test for comparison between categorical variables, and a p-value of < 0.05 was considered statistically significant.

Results

Of the 1223 specimens, 1020 specimens were culture positive, with 240 (23.52%) yielding MDROs, with the highest proportions isolated from peritoneal fluid, CSF, and pus. *Escherichia coli* was the most common MDRO, with 73 (30.4%) isolates, followed by 37 (15.4%) isolates of *Klebsiella* spp., 29 (12.1%) isolates of *Pseudomonas* spp., and 19 (7.9%) isolates of *Acinetobacter* spp. The majority of MDROs were isolated from general wards, with 112 (46.7%) MDROs rather than 47 (19.6%) MDROs from the critical care unit.

Conclusions

This study highlights the significant burden of MDROs in a tertiary care center, with a high proportion of resistance to last-line agents. The findings emphasize the need for regular surveillance, strict adherence to infection control practices, and effective antimicrobial stewardship programs to mitigate the growing MDRO burden. Future recommendations include establishing robust antimicrobial stewardship programs, conducting periodic hospital-specific antibiogram reviews, and promoting research on emerging resistance mechanisms.

## Introduction

Multidrug-resistant organisms (MDROs) are usually defined as microorganisms that are nonsusceptible to at least one agent in three or more antimicrobial classes, as proposed in standard international definitions [1]. MDROs in hospitals and the community pose a major global public health threat [1]. Irrational antibiotic use, over-the-counter access to antimicrobials, empirical prescribing without culture guidance, and lack of prescription audit systems have driven the rapid emergence of MDROs [2,3], while the antibiotic pipeline has simultaneously stagnated, with pharmaceutical companies failing to introduce sufficient new agents [4]. Selecting an appropriate empirical antimicrobial has therefore become increasingly challenging, underscoring the need for regular, locally generated antibiograms to guide therapy, stewardship interventions, and treatment protocols [5].

MDROs of key concern include methicillin-resistant *Staphylococcus aureus*, vancomycin-resistant enterococci, and multidrug-resistant (MDR) gram-negative bacilli (GNB), such as extended-spectrum β-lactamase (ESBL)-producing strains [6,7]. Important GNB include *Escherichia coli*, *Klebsiella pneumoniae*, *Acinetobacter baumannii *[5], *Stenotrophomonas maltophilia *[5], *Burkholderia cepacia *[8], and *Ralstonia pickettii *[9], many of which are intrinsically resistant to broad-spectrum agents and are frequently implicated in healthcare-associated infections [10]. Although transmission is most clearly documented in acute-care hospitals, virtually all healthcare settings, including intensive care units, step-down units, and general wards, are affected by the emergence and spread of these organisms. The clinical impact varies across institutions and populations, but MDRO infections consistently increase length of stay, cost of care, and mortality [11,12]. Outbreaks of ESBL-producing *K. pneumoniae *in neonatal intensive care units and the emergence of third-generation cephalosporin-resistant *Enterobacter *spp. in adults highlight the severe consequences of MDR-GNB [6,13].

Resistance patterns vary across hospitals, units, specimen types, and patient populations. Therefore, hospital-specific surveillance is essential for guiding empirical therapy, infection prevention strategies, and antimicrobial stewardship. Data from our region remain limited, especially regarding the distribution of MDROs across different clinical specimens and care areas. In this context, the present study was undertaken to characterize the spectrum of MDROs isolated in a tertiary care center, describe their distribution across clinical specimens and hospital care areas, identify associated risk factors, and summarize their antimicrobial susceptibility patterns.

## Materials and methods

Study design and setting

This prospective observational study was conducted over a period of one month in the Department of Microbiology of a tertiary care center in Northern India. All eligible clinical specimens received in the bacteriology section from predefined hospital areas during the study period were processed for culture, identification, and antimicrobial susceptibility testing (AST).

Study population

All clinical samples from defined hospital areas received in the bacteriology section of the microbiology department for identification and AST profiling during the study duration were included. Wherever feasible, only specimens collected before initiation of antimicrobial therapy were included. Samples obtained immediately after commencement of antibiotic treatment were excluded from analysis. A total of 1223 clinical specimens received during the study period were processed. Samples showing significant bacterial or fungal growth were further evaluated, and isolates fulfilling the study definition of MDRO were included in the final analysis.

Data collection

For each enrolled sample, patient demographic details and clinical information were recorded. The following risk factors were assessed: immunocompromised status, prolonged hospital stay or ICU stay, prior intake of antibiotics and duration of use, presence of significant comorbidities, and use of interventional medical devices or implants. Infection prevention and control (IPC)-related information was documented for the included study areas.

Sample collection and processing

All samples were collected under aseptic precautions and processed according to standard microbiological procedures:

Direct Microscopy

Gram staining was performed for pus, sputum, blood, body fluids, and swabs. Smears were examined for pus cells and microorganisms. Sputum and pleural fluid were graded as per Bartlett’s classification. For blood cultures, smears were prepared directly from bottles flagged positive by the BACT/ALERT 3D system (bioMérieux, Salt Lake City, UT, USA).

Urine

Midstream clean-catch urine samples were collected in sterile, dry, leak-proof screw-capped containers after proper patient instruction and processed within two hours. Wet mounts were examined for pus cells, red blood cells, and microorganisms. Semi-quantitative culture was performed by inoculating a standard loopful (4 mm) of well-mixed urine onto cystine-lactose-electrolyte-deficient agar and incubating at 37°C for 18-24 hours.

Pus and Wound Swabs

Pus was collected in a syringe or on sterile swabs and directly inoculated onto blood agar and MacConkey agar, with a small portion inoculated into brain-heart infusion (BHI) broth. Plates were incubated at 37°C for 18-24 hours. If no growth occurred on solid media but BHI showed turbidity, subculture was performed. Smears were Gram stained to assess pus cells and organisms.

Respiratory Tract Samples

Sputum was collected in wide-neck sterile containers. Smears from the purulent portion were Gram stained; samples with >25 pus cells and <10 epithelial cells per high-power field were processed for aerobic culture. Endotracheal tube tips received in sterile containers were rolled over culture media for inoculation. Bronchoalveolar lavage (BAL) and gastric aspirate were collected aseptically, Gram stained, and cultured on blood agar, MacConkey agar, and chocolate agar. All plates were incubated at 37°C for 18-24 hours.

Blood

Two blood samples from different venipuncture sites were inoculated into BacT/ALERT 3D automated culture bottles under aseptic conditions. Bottles flagged positive were inspected for turbidity and subcultured onto blood agar and MacConkey agar, followed by incubation at 37°C for 18-24 hours.

Sterile Body Fluids

CSF and other sterile fluids were collected in sterile, leak-proof containers and transported to the laboratory within two hours. Triple-layer smears were Gram stained. Samples were inoculated onto blood agar, MacConkey agar, and chocolate agar and into BHI broth. Plates were incubated at 37°C for 18-24 hours; turbid BHI with no initial plate growth was subcultured. Absence of growth in subculture was considered indicative of sterility.

Identification and AST

Colonies grown on characteristic media were identified by Gram stain and standard biochemical/phenotypic criteria, followed by confirmation and AST using automated systems: MALDI-TOF MS for identification and VITEK 2 (bioMérieux) for antibiotic susceptibility testing. Isolates were classified as MDR when they showed non-susceptibility to at least one agent in three or more antimicrobial classes, in accordance with standard international definitions.

Statistical analysis

Data were entered in Microsoft Excel (Microsoft Corporation, Redmond, WA, USA) and analyzed using IBM SPSS Statistics for Windows, version 22.0 (released 2013; IBM Corp., Armonk, NY, USA). Categorical variables were summarized as frequencies and percentages. The chi-square test was used for comparison of categorical variables where applicable. A p-value < 0.05 was considered statistically significant.

## Results

Of the 1223 clinical specimens processed (the maximum number of urine samples were received, followed by sputum, blood, pus, and sterile body fluids), 240 (19.62%) yielded MDROs. The highest proportions of MDROs were seen in peritoneal fluid (1; 50%) isolates, CSF (14; 48.27%) isolates, and pus (64; 35.5%) isolates, followed by ascitic fluid with 6 (30%) isolates, pleural fluid with 7 (24.13%) isolates, BAL with 3 (21.42%) isolates, and sputum with 39 (18.75%) isolates. Urine, although the most frequently received specimen, showed a lower MDRO rate, with only 89 (16.03%) out of 555 received isolates, and blood had the lowest proportion among positive categories, with 17 (9.23%) isolates. No MDROs were isolated from gastric aspirate (Table 1).

**Table 1:** Percentage of MDROs from different clinical specimens BAL, bronchoalveolar lavage; GA, gastric aspirate; MDROs, multidrug-resistant organisms

Specimen	Total received	Total MDROs grown	% of MDROs
Urine	555	89	16.03%
Sputum	208	39	18.75%
Blood	184	17	9.23%
Pus	180	64	35.5%
CSF	29	14	48.27%
Pleural fluid	29	7	24.13%
Ascitic fluid	20	6	30.0%
BAL	14	3	21.42%
GA	2	0	0%
Peritoneal fluid	2	1	50%
Total	1223	240	19.62%

Among the 240 (19.62%) MDRO isolates, *E. coli *was the most common, with 73 (30.4%) isolates, followed by *Klebsiella *spp. with 37 (15.4%) isolates, *Pseudomonas *spp. with 29 (12.1%) isolates, and *Acinetobacter *spp. with 19 (7.9%) isolates. Urine MDROs were predominantly *E. coli *and *Klebsiella *spp., whereas pus showed a mixed profile with *S. aureus*, *E. coli*, *Pseudomonas *spp., and *Klebsiella *spp. Respiratory samples (sputum and BAL) were mainly infected with *Pseudomonas *spp., *Klebsiella *spp., *E. coli*, *Acinetobacter *spp., and coagulase-negative staphylococci (CoNS). Blood isolates were chiefly CoNS and *Salmonella *spp., while CSF and pleural fluid were dominated by non-fermenters such as *Acinetobacter *spp., *Pseudomonas *spp., and *Enterobacter* spp. Overall, GNB, particularly *E. coli *and *Klebsiella *spp., formed the major burden of MDROs across clinical specimens (Table 2).

**Table 2:** Distribution of MDROs in different clinical samples BAL, bronchoalveolar lavage; CoNS, coagulase-negative staphylococci; MDROs, multidrug-resistant organisms

Growth of MDROs in various clinical specimens	*Pseudomonas *spp.	*Acinetobacter *spp.	*Enterococcus *spp.	*Enterobacter *spp.	*Achromobacter *spp.	Escherichia coli	Staphylococcus aureus	CoNS	*Streptococcus *spp.	*Klebsiella *spp.	Serratia marcescens	*Aeromonas *spp.	Candida albicans	Citrobacter koseri	*Salmonella *spp.
Urine (89)	3	7	4	-	-	53	-	4	-	14	-	-	3	-	1
Pus (64)	9	3	5	4	-	9	14	7	2	7	1	1	1	1	-
Sputum (39)	12	3	-	2	-	8	-	-	-	10	-	1	2	1	-
Blood (17)	1	1	1	-	1	1	-	6	-	-	-	-	2	-	4
CSF (14)	3	4	-	4	-	-	-	1	-	2	-	-	-	-	-
Pleural fluid (7)	1	-	-	4	-	-	-	-	-	2	-	-	-	-	-
Ascitic fluid (6)	-	-	2	-	-	1	-	1	-	2	-	-	-	-	-
Peritoneal fluid (1)	-	-	-	-	-	1	-	-	-	-	-	-	-	-	-
BAL (3)	-	1	-	-	-	-	-	2	-	-	-	-	-	-	-
Total (240)	29	19	12	14	1	73	14	21	2	37	1	2	8	2	5

**Figure 1:**
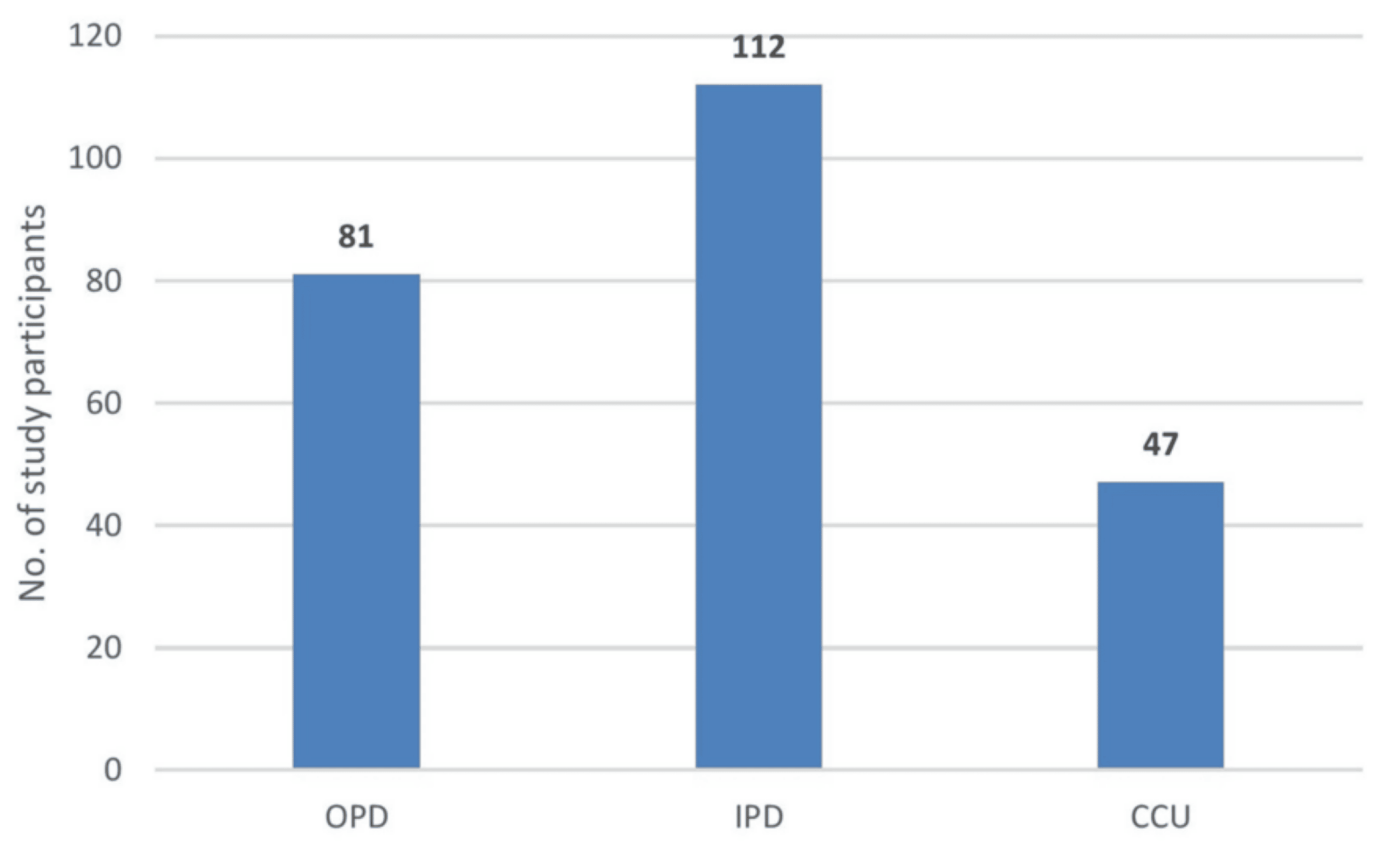
OPD/IPD/CCU-wise MDRO distribution CCU, critical care unit; IPD, inpatient department; MDROs, multidrug-resistant organisms; OPD, outpatient department

MDROs were most frequently isolated from inpatients, 112 (46.7%), followed by 81 (33.75%) OPD cases, with the lowest numbers from CCU, 47 (19.6%), indicating that the main burden of MDROs in this cohort lay in the general wards rather than the ICU (Figure 1).

Immunocompromised status was seen in 117 (48.75%) patients. A total of 154 (64.16%) patients had a history of prolonged hospital or ICU stay. Additionally, 150 (62.5%) patients had already taken antibiotics before samples were sent for culture and sensitivity. Only 68 (28.33%) patients had comorbid conditions, and only 66 (27.5%) patients had a history of device intervention.

**Figure 2:**
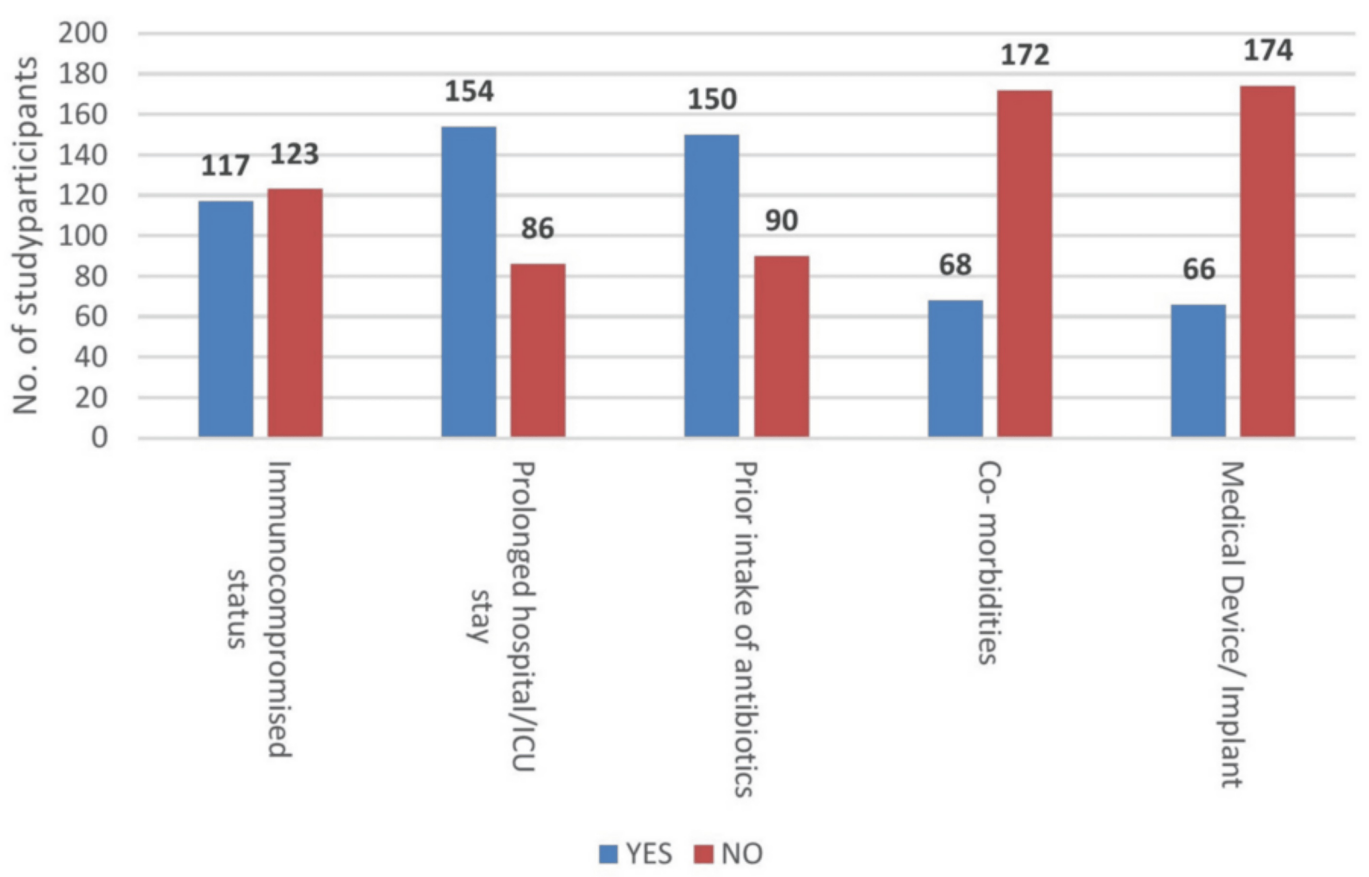
Percentages of risk factors

Prior healthcare exposure was an important contributor to MDRO acquisition. A clear majority of patients had a history of prolonged hospital/ICU stay (154, 64.16% vs 86, 35.83%) and prior antibiotic use (150, 62.5% vs 90, 37.5%), whereas only about half were immunocompromised (117, 48.75% vs 123, 51.25%). Most did not have associated comorbidities or indwelling medical devices/implants, implying that length of stay and antimicrobial exposure are stronger drivers than baseline illness or device use in this group (Figure 2).

**Figure 3:**
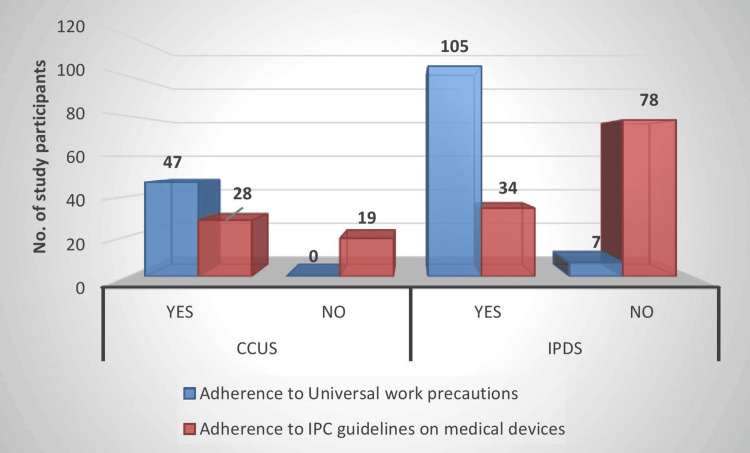
Percentage of IPC practices followed in CCUs and wards CCU, critical care unit; IPC, infection prevention and control; IPD, inpatient department

Figure 3 demonstrates generally good adherence to universal work precautions in both CCUs (47, 100%) and IPDs (105, 93.75%), but suboptimal adherence to IPC guidelines for medical devices, particularly in the wards (only 34, 30.35% compliant vs 78, 69.64% non-compliant), highlighting device-related practices in IPDs as a potential area for targeted infection-prevention interventions.

Out of 240 (19.62%) MDRO isolates subjected to antibiotic sensitivity testing by the VITEK 2 automated method, *E. coli *was the predominant pathogen, showing resistance mainly to colistin, followed by *Klebsiella *spp., which was predominantly resistant to imipenem, followed by gentamicin. *Pseudomonas *spp. was mostly resistant to imipenem, followed by levofloxacin. *Acinetobacter *spp. was mostly resistant to imipenem, followed by meropenem, except for minocycline. Among critical care units (CCUs), the highest burden of MDROs was seen with *Achromobacter *spp. and *Serratia *spp., followed by *Enterococcus *spp. On the other hand, *Salmonella *spp. showed the highest burden in inpatient wards, followed by *Candida albicans *infection.

Among the 240 (19.62%) MDRO isolates, 112 (46.66%) were from IPD wards and 47 (19.58%) were from the CCU. Ward-based infections predominated for most organisms, particularly *Salmonella *spp. with 5 (100% IPD) isolates, *C. albicans *with 6 (75%) isolates, *Klebsiella *spp. with 19 (51.35%) isolates, *Pseudomonas *spp. with 18 (62.06%) isolates, *Enterobacter *spp. with 9 (64.28%) isolates and *Acinetobacter *spp. with 12 (63.15%) isolates. In contrast, certain pathogens were isolated exclusively or proportionately more from the CCU, such as *Achromobacter *spp. with 1 (100% CCU) isolate and *Serratia *spp. with 1 (100% CCU) isolate, while *Enterococcus *spp. showed an equal distribution between IPD and CCU with 5 (41.66%) isolates each. Notably, *E. coli*, the most frequent MDRO overall, accounted for 20 (27.39%) of IPD isolates and 10 (13.69%) of CCU isolates, indicating a substantial burden in both settings (Table 3).

**Table 3:** Correlation of MDRO burden in CCU and wards CCU, critical care unit; CoNS, coagulase-negative staphylococci; IPD, inpatient department; MDROs, multidrug-resistant organisms

MDROs	% in IPD	% in CCU
*Achromobacter*spp. (n = 1)	-	1 (100%)
*Acinetobacter *spp. (n = 19)	12 (63.15%)	6 (31.5%)
*Aeromonas*spp. (n = 2)	1 (50%)	-
*Candida albicans*(n = 8)	6 (75%)	2 (25%)
*Citrobacter koseri*(n = 2)	-	-
*Escherichia coli*(n = 73)	20 (27.39%)	10 (13.69%)
*Enterobacter*spp. (n = 14)	9 (64.28%)	3 (21.42%)
*Enterococcus* spp. (n = 12)	5 (41.66%)	5 (41.66%)
*Klebsiella* spp. (n = 37)	19 (51.35%)	10 (27.02%)
*Pseudomonas* spp. (n = 29)	18 (62.06%)	7 (24.13%)
*Salmonella* spp. (n = 5)	5 (100%)	-
*Serratia marcescens* (n = 1)	-	1 (100%)
*Staphylococcus aureus* (n = 14)	4 (28.57%)	-
CoNS (n = 21)	12 (5.14%)	2 (9.5%)
*Streptococcus* spp. (n = 2)	1 (50%)	-
Total (n = 240)	112 (46.66%)	47 (19.58%)

## Discussion

In our study, 19.62% of culture-positive clinical specimens yielded MDROs, with the highest proportions isolated from peritoneal fluid, CSF, and pus, while urine, though the most frequently received specimen, showed a comparatively lower MDRO rate. This pattern differs from the findings of Pattnaik et al., who reported an overall MDR prevalence of 66.1% and a predominance of MDR isolates in urine and respiratory samples, reflecting a very high burden of resistant GNB in routine specimens from inpatients [14]. Similarly, Namdari et al. observed that Gram-negative infections were largely dominated by urinary tract infections (over 60%), again with *E. coli *as the principal pathogen [15]. Our data, in contrast, highlight that while urine is the most common sample, the proportion of MDROs is particularly high in normally sterile body fluids, underlining the seriousness of invasive MDRO infections in our setting.

The distribution of organisms in our study is broadly in line with other reports. We found *E. coli *(30.4%) and *Klebsiella *spp. (15.4%) to be the predominant MDROs, followed by *Pseudomonas *and *Acinetobacter*, similar to Pattnaik et al. and Namdari et al., who also reported Enterobacterales and non-fermenters as the main contributors to the MDR burden [14,15]. However, Singh et al. reported *K. pneumoniae *as the leading MDRO, followed by *A. baumannii *and *E. coli*, suggesting that local antibiograms and ecological pressures can shift the dominant species even within tertiary-care settings [16]. The high MDRO burden of *Salmonella *spp. and *C. albicans *in our wards also indicates substantial pressure on both enteric bacterial and opportunistic fungal pathogens, which is less prominently described in the comparative studies.

A striking finding in our cohort was that the majority of MDROs were isolated from general wards (46.7%) rather than the CCU (19.6%), despite the conventional expectation of higher resistance in ICUs. Pattnaik et al. documented significantly higher MDR and extensively drug-resistant (XDR) rates in ICUs compared with clinical wards [14], and Singh et al. also noted high MDRO loads in high-dependency departments such as general medicine and surgery [16]. In our setting, better adherence to universal work precautions in CCUs contrasted with suboptimal device-related infection prevention practices in IPDs, which may partly explain the relatively higher ward burden. This suggests that, where ICU-level infection control is rigorously enforced, general wards may become the “weak link” for MDRO propagation, particularly when device care and surveillance are less stringent.

The risk factor profile in our study reinforces the central role of healthcare exposure and antibiotic pressure in MDRO acquisition. Nearly two-thirds of patients had a history of prolonged hospital/ICU stay and prior antibiotic use, whereas only about half were immunocompromised, and less than one-third had comorbidities or invasive devices. This aligns with broader AMR literature and with findings from Singh et al., who observed a surge in MDROs in the post-COVID-19 period, likely driven by heavy antimicrobial use and prolonged hospitalization [16]. These patterns are consistent with earlier global observations that emphasize antibiotic misuse and healthcare contact as critical drivers of resistance, sometimes more important than baseline host factors [17-19].

The antibiotic resistance profile in our isolates shows both parallels and worrying divergences from other studies. We found very high resistance to colistin among *E. coli *(79%), which is particularly alarming because colistin is traditionally reserved as a last-line agent. In contrast, Namdari et al. reported preserved susceptibility of *Acinetobacter *and other non-fermenters to colistin, and Al-Hasani et al. observed good sensitivity of *E. coli *to carbapenems and piperacillin-tazobactam, despite extensive resistance to older β-lactams and fluoroquinolones [7,15]. Our Klebsiella isolates showed marked resistance to imipenem and gentamicin, while *Acinetobacter *exhibited high carbapenem resistance but retained susceptibility to minocycline, patterns broadly concordant with Pattnaik et al., who documented very high MDR and XDR rates among *Klebsiella *and *Acinetobacter*, with a small but worrying proportion of pandrug-resistant strains [14]. Taken together, these comparisons suggest that our institution is facing a more advanced stage of resistance to last-resort agents, especially colistin among Enterobacterales, which may reflect intensive use of these drugs in critically ill patients and insufficient stewardship.

Regarding bloodstream infections, we observed MDROs in a smaller proportion of positive blood cultures (9.23%), mainly CoNS, *Salmonella*, and non-fermenters, whereas Vihari et al. reported a substantial burden of Gram-positive BSIs with high rates of resistant CoNS and *Enterococcus *spp. and a mortality exceeding 40% [20]. While our study was not focused specifically on BSIs or outcomes, the concordance in pathogen profile (CoNS, *Enterococcus*, and resistant *Staphylococci*) underscores that resistant Gram-positive organisms remain important contributors to severe sepsis, particularly in high-risk settings.

Antimicrobial resistance in hospital pathogens is largely driven by inappropriate antibiotic use, ICU selective pressure, and rapid plasmid-mediated gene transfer, such as the *mcr *gene in Enterobacteriaceae [21]. Carbapenem resistance in *Klebsiella *spp. commonly results from carbapenemase production (KPC, NDM, and OXA-48) and aminoglycoside-modifying enzymes [21]. Intrinsic mechanisms in *Pseudomonas *spp., including efflux pumps, porin loss, and biofilm formation, contribute to resistance to carbapenems and fluoroquinolones [22]. *Acinetobacter *spp. persist in hospital environments due to their ability to acquire carbapenem-hydrolyzing oxacillinases, though minocycline may remain effective [22]. Higher MDRO rates in CCUs worldwide are associated with prolonged hospitalization, invasive devices, broad-spectrum antibiotic exposure, and cross-transmission. Device-associated infections and selective pressure from cephalosporins and glycopeptides also promote resistance in organisms such as *Enterococcus *spp. [23].

Overall, comparison with other studies shows that our institution shares the global pattern of MDRO predominance by *E. coli*, *Klebsiella *spp., and *Acinetobacter *spp., but with some distinct features: (1) a particularly high proportion of MDROs in sterile body fluids; (2) a shift of major MDRO burden toward general wards rather than ICUs; and (3) very high resistance to last-line agents such as colistin in Enterobacterales. These differences underline the need for hospital-specific surveillance, ward-focused infection control strengthening, and aggressive antimicrobial stewardship tailored to our local resistance ecology.

Limitations

The present study has several limitations. It was conducted over a short duration of one month in a single tertiary care center, which limits the generalizability of the findings and may not adequately capture seasonal or temporal variation in antimicrobial resistance patterns. Although 1223 clinical samples were processed, only 240 MDRO isolates were identified, and a larger number of isolates or a longer surveillance period would have improved the precision and representativeness of the findings. The study was primarily descriptive in nature, and comparisons across hospital areas such as wards and CCUs were not normalized to patient days or admission load. Molecular confirmation of resistance mechanisms, including detection of genes such as NDM, KPC, or *mcr*, was not performed due to time and financial constraints; similarly, target sequencing could not be undertaken, which might have provided deeper insight into the underlying resistance mechanisms. In addition, colistin resistance detected by the automated system was not confirmed by broth microdilution. The possibility of contamination or colonization could not be completely excluded for some clinical specimens. The study also did not include assessment of MDROs in livestock-associated bacterial isolates, which might have provided a broader understanding of the resistance landscape. Furthermore, adherence to IPC measures was suboptimal in some areas, with lower adherence observed in IPDs than in CCUs, suggesting scope for stricter training and compliance protocols. Multicentric studies with longer surveillance periods, larger isolate numbers, molecular characterization, and broader ecological assessment would provide a more comprehensive understanding of the local and regional MDRO burden.

## Conclusions

The study highlights that MDROs pose a major threat in the institute, acting as important opportunistic pathogens with resistance to commonly used antibiotics. Regular speciation and continuous surveillance of antimicrobial susceptibility patterns are essential, as pathogen prevalence and resistance profiles vary across hospitals and patient populations. Clinical microbiologists must consistently update clinicians on circulating pathogens to guide rational empirical therapy. Strict adherence to infection control practices, including isolation protocols, hand hygiene, use of personal protective equipment, environmental cleaning, and proper sterilization of instruments, remains critical, as MDROs can persist in hospital environments. Effective biomedical waste management further reduces transmission risk.

Hospitals should strengthen antimicrobial stewardship activities, develop periodic antibiograms, improve adherence to infection prevention protocols, and promote regular staff training. Future research should include longer surveillance periods, multicentric participation, and molecular characterization of resistance mechanisms to provide a more comprehensive understanding of the evolving MDRO landscape.

## References

[1] Magiorakos AP, Srinivasan A, Carey RB (2012). Multidrug-resistant, extensively drug-resistant and pandrug-resistant bacteria: an international expert proposal for interim standard definitions for acquired resistance. Clin Microbiol Infect.

[2] Gandra S, Tseng KK, Arora A (2019). The mortality burden of multidrug-resistant pathogens in India: a retrospective, observational study. Clin Infect Dis.

[3] Hashemi S, Nasrollah A, Rajabi M (2013). Irrational antibiotic prescribing: a local issue or global concern?. EXCLI J.

[4] Dhanda G, Acharya Y, Haldar J (2023). Antibiotic adjuvants: a versatile approach to combat antibiotic resistance. ACS Omega.

[5] Ruiz-Ramos J, Escolà-Vergé L, Monje-López ÁE, Herrera-Mateo S, Rivera A (2023). The interventions and challenges of antimicrobial stewardship in the emergency department. Antibiotics (Basel).

[6] Siegel JD, Rhinehart E, Jackson M, Chiarello L (2006). Management of multidrug-resistant organisms in healthcare settings, 2006. https://www.cdc.gov/infection-control/media/pdfs/guideline-mdro-h.pdf.

[7] Al-Hasani HM, Al-Rubaye DS, Abdelhameed A (2023). The emergence of multidrug-resistant (MDR), extensively drug-resistant (XDR), and pandrug-resistant (PDR) in Iraqi clinical isolates of Escherichia coli. J Popul Ther Clin Pharmacol.

[8] Häfliger E, Atkinson A, Marschall J (2020). Systematic review of healthcare-associated Burkholderia cepacia complex outbreaks: presentation, causes and outbreak control. Infect Prev Pract.

[9] Basso M, Venditti C, Raponi G (2019). A case of persistent bacteraemia by Ralstonia mannitolilytica and Ralstonia pickettii in an intensive care unit. Infect Drug Resist.

[10] Bharadwaj A, Rastogi A, Pandey S, Gupta S, Sohal JS (2022). Multidrug-resistant bacteria: their mechanism of action and prophylaxis. Biomed Res Int.

[11] Cohen CC, Cohen B, Shang J (2015). Effectiveness of contact precautions against multidrug-resistant organism transmission in acute care: a systematic review of the literature. J Hosp Infect.

[12] Tajeddin E, Rashidan M, Razaghi M (2016). The role of the intensive care unit environment and health-care workers in the transmission of bacteria associated with hospital acquired infections. J Infect Public Health.

[13] Banerjee T, Bhattacharjee A, Upadhyay S (2016). Long-term outbreak of Klebsiella pneumoniae & third generation cephalosporin use in a neonatal intensive care unit in north India. Indian J Med Res.

[14] Pattnaik D, Panda SS, Singh N (2019). Multidrug resistant, extensively drug resistant and pan drug resistant gram negative bacteria at a tertiary care centre in Bhubaneswar. Int J Community Med Public Health.

[15] Namdari S, Farhadi A, Khademalhoseini A, Behzad-Behbahani A, Moaddeb A (2021). Emergence of highly multidrug-resistant bacteria isolated from patients with infections admitted to public hospitals in Southwest Iran. Interdiscip Perspect Infect Dis.

[16] Singh H, Patel AA, Pandy P (2025). Burden of multi-drug-resistant organisms in a tertiary healthcare institute in North India: implications for regional public health. World J Exp Med.

[17] Roca I, Akova M, Baquero F (2015). The global threat of antimicrobial resistance: science for intervention. New Microbes New Infect.

[18] Luo G, Lin L, Ibrahim AS (2012). Active and passive immunization protects against lethal, extreme drug resistant-Acinetobacter baumannii infection. PLoS ONE.

[19] Mba IE, Sharndama HC, Anyaegbunam ZK (2023). Vaccine development for bacterial pathogens: advances, challenges and prospects. Trop Med Int Health.

[20] Vihari N, Bohra GK, Yadev RR (2023). The emergence of multidrug-resistant Gram-positive bloodstream infections in India—a single center prospective cohort study. Germs.

[21] Karampatakis T, Tsergouli K, Behzadi P (2023). Carbapenem-resistant Klebsiella pneumoniae: virulence factors, molecular epidemiology and latest updates in treatment options. Antibiotics (Basel).

[22] Velmurugan P, Ramalingam AJ, Saikumar C (2024). An ancient drug for a modern era: minocycline for the treatment of multi-drug-resistant Acinetobacter baumannii. Cureus.

[23] Rao C, Dhawan B, Vishnubhatla S, Kapil A, Das B, Sood S (2020). Emergence of high-risk multidrug-resistant Enterococcus faecalis CC2 (ST181) and CC87 (ST28) causing healthcare-associated infections in India. Infect Genet Evol.

